# One-time low concentration betadine eye wash: A novel treatment for epidemic viral conjunctivitis for accelerated recovery

**DOI:** 10.22336/rjo.2024.49

**Published:** 2024

**Authors:** Sumedha Vats, Anchal Tripathi, Inam Danish Khan, Pawan Dhull, Sanjay Kumar Mishra, Ranjit Goenka, Devendra Paul Vats

**Affiliations:** 1 Department of Ophthalmology, Armed Forces Clinic, New Delhi, India; 2 Department of Ophthalmology, Military Hospital, Jammu, India; 3 Department of Clinical Microbiology and Infectious Diseases, Armed Forces Clinic, New Delhi, India; 4 DNB Neurology, Armed Forces Clinic, New Delhi, India; 5 Department of Ophthalmology, Army Hospital Research and Referral, New Delhi, India; 6 Department of Ophthalmology, Base Hospital Delhi Cantt, New Delhi, India; 7 Maharaja Agrasen Medical College, Agroha, India

**Keywords:** epidemic conjunctivitis, viral conjunctivitis, adenovirus, enterovirus, staphylococcus, betadine wash

## Abstract

**Purpose:**

This study aims to renew the management of viral epidemic conjunctivitis by introducing a one-time, low-concentration ocular surface povidone-iodine (LOS-pI) wash.

**Methods:**

Among the 3,002 patients screened, 1,328 with acute conjunctivitis were categorized into two groups. Group A (664 patients) underwent a 1% betadine wash in addition to the standard treatment protocol (Eye Lubricant + Moxifloxacin 0.5% eyedrops), while Group B (664 patients) followed the standard protocol alone. In cases of membranous conjunctivitis, manual membrane removal was performed. Treatment responses were observed daily for three days, followed by weekly assessments for two additional weeks.

**Results:**

Co-infection of adenovirus with enterovirus was found to be the main cause, often accompanied by staphylococcal superinfection. Group A showed complete resolution of conjunctival inflammation, with a remarkable 76.05% of patients experiencing improvement within an average of 2.6±0.51 days, in contrast to Group B’s average of 7.5±1.1 days (p <0.05). Additionally, 13% of Group B patients with recalcitrant conjunctivitis significantly recovered following the 1% betadine wash. Complications (subconjunctival hemorrhage: 34.04%, superficial punctate keratitis: 6.02%) were more prevalent in Group B.

**Discussion:**

The authors hypothesized that a single wash with betadine is sufficient to reduce disease duration and prevent secondary infections and complications. The core strength of our study lies in its substantial sample size. To our knowledge, no similar previous research has been conducted, on such a larger scale.

**Conclusion:**

Viral conjunctivitis brings discomfort, work absenteeism, and financial burden. A single low-concentration betadine wash expedites recovery and reduces complications in acute infective conjunctivitis. This approach significantly enhances patient outcomes and alleviates the socioeconomic impact of the condition.

## Introduction

Conjunctivitis ranks amongst the most prevalent conditions in an ophthalmology outpatient department [1]. It is a source of great patient discomfort, leading to absence from work, reduced productivity, and financial loss [2]. Any inflammation of the conjunctiva, irrespective of the etiology, is termed conjunctivitis, the most common cause of which remains viral (adenoviral) infections [3]. Allergic, immune-mediated, mechanical, toxic, neoplastic, etc., are non-infectious causes of conjunctivitis [4]. Based on the onset, conjunctivitis is also categorized as, hyperacute, acute, and chronic [5]. Viral and bacterial conjunctivitis are often acute in presentation. However, such cases might present occasionally as chronic infections, which means they last for more than three to four weeks [6]. Not only does this affect the health of the patient’s ocular surface, but, it also leads to a significant economic burden [2]. Inflammation due to viral or bacterial infections can, sometimes, lead to membranous or pseudomembranous conjunctivitis [6]. In such cases, the fibrin layer formation and other inflammatory deposits over the palpebral conjunctiva occur [6]. The difference between membranous and pseudomembranous conjunctivitis is that the true membrane formation occurs in membranous conjunctivitis, and its manual removal results in bleeding [6]. Early treatment in all such cases can result in the prevention of complications and reduced economic burden.

Epidemics of viral conjunctivitis, as witnessed in most parts of India in July 2023, are commonly caused by adenovirus [7]. The contagiousness of this infection is so high that a single infected individual can transmit the disease to a whole family of as many as seven people, and even more. Resolution of symptoms and resumption to normalcy may take several days to weeks [7]. No treatment for viral conjunctivitis exists at present, the management lies in the self-resolution along with the prevention of secondary infections, and management of complications, as they appear [7].

In this study, we aimed to evaluate the efficacy of one-time day-care low-concentration ocular surface povidone-iodine (LOS-pI) wash as an adjuvant for cases of recalcitrant infectious conjunctivitis.

## Methods

This study was conducted at a tertiary eye care hospital in Northern India, from June 2023 to August 2023. A total of 3002 patients, having red eye, were screened. Out of these, 1328 patients with acute conjunctivitis were recruited. Of these, 664 patients (Group A) received 1% betadine wash one time along with the treatment protocol, meaning lubricant eyedrops and moxifloxacin 0.5% eyedrops (both four times per day). This group served as a case group. Other 664 patients (Group B) were managed per protocol without betadine and served as the control group (**Table 1**).

**Table 1:** Comparison of demographic variables between Group A and Group B

Parameters	Group GROUPA (n=664)	GROUPB (n=664)	p-value
**Age(Years)**	29.10±7.04	29.16±7.27	>0.5^1^
**Gender**			>0.1^2^
Male	29.8%	41.6%	
Female	15.9%	12.7%	

*** Significant at p < 0.05, 1: Wilcoxon-Mann-Whitney U Test, 2: Chi-Squared Test

The Institutional ethical committee approved this study, per the tenets of the Declaration of Helsinki. Written informed consent was obtained from all the patients, and all the data was anonymized and double-blinded.

All the patients underwent complete ophthalmological examination, to rule out other causes of ocular surface inflammation. Patients having hypo/hyperthyroidism, iodine allergy, or any other cause of ocular inflammation, were excluded from the study.

Clinical suspicion of Staphylococcus aureus for follicular conjunctivitis, and adenovirus for hemorrhagic conjunctivitis was confirmed by microbiological diagnosis for selected cases. Plain sterile cotton conjunctival swabs were obtained from the palpebral conjunctiva of the affected eye for bacterial microscopy and culture. Simultaneously, Dacron conjunctival swabs in a viral transport medium were obtained to establish viral etiology. Bacteriological routine methods such as Gram stain and culture were followed by automated identification and susceptibility through the Vitek-2 compact system (Biomerieux, France). The virological diagnosis was established by a Biofire multiplex film array targeting multiple viruses simultaneously (Biomerieux, France). Post-analytical interpretation of selected patients was extrapolated to all patients with a similar clinical presentation.

### The procedure of one-time day-care low-concentration ocular surface povidone-iodine (LOS-pI) wash

**Fig. 1:**
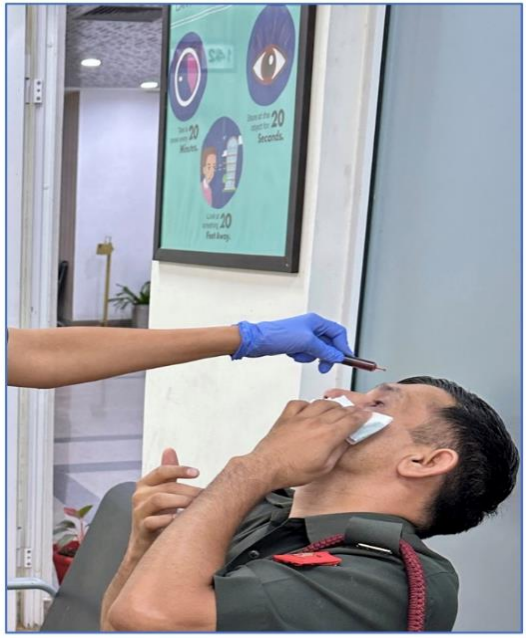
Procedure of one-time, low-concentration ocular surface povidone-iodine (LOS-pI) wash in the seated position of the patient

**Fig. 2:**
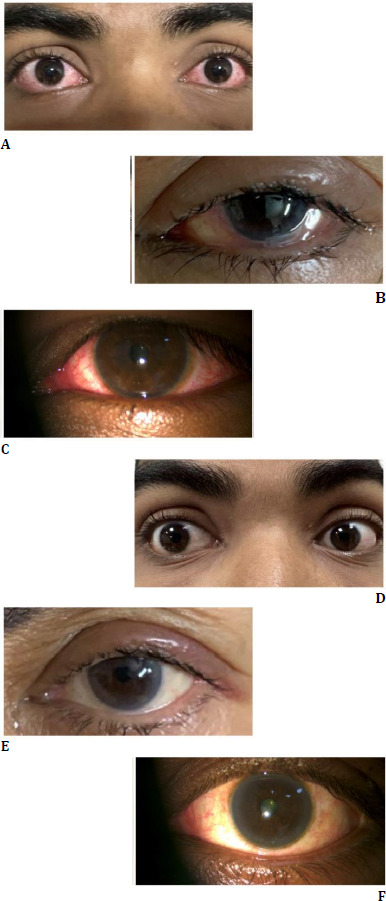
**A-C** Group A patients on the day of presentation; **D-F**. Group A patients three days after treatment with one-time, low-concentration ocular surface povidone-iodine (LOS-pI) wash. A remarkable reduction in conjunctival injection, lacrimation, discharge, and lid edema is observed

Betadine is commonly available as a 5% and a 10% solution. We used a 10% betadine solution. 1 mL of this 10% solution was diluted in 10 mL normal saline, to make 1% of betadine solution (low concentration). While the patients were seated, the lower and upper fornices were washed with the solution, under topical anesthesia and strict aseptic precautions (**Fig. 1**). It was followed by a gentle saline wash after two minutes. Safety and tolerability of LOS-pI wash were assessed by corneal fluorescein staining/visual acuity and LOS-pI wash associated ocular discomfort, respectively, which were determined at baseline, on the day of LOS-pI wash, immediately post LOS-pI wash, and on day one of LOS-pI wash (**Fig. 2**).

### Outcome measures and follow-up

The conjunctival signs of inflammation, meaning the conjunctival injection grade, were noted at each follow-up. The patient satisfaction level was measured on a scale of five satisfaction levels, along with symptom reduction such as ocular discomfort/mild ocular pain, foreign body sensation/grittiness, redness, discharge/watering, photophobia, and lid edema. The infection was “completely resolved” if the grade of conjunctival injection was reduced to zero, along with a reduction of all the symptoms to zero, and an increment of patient satisfaction level to five. No microbiological/virological follow-up was done. This management was considered “treatment failure” in case of less than a two-step reduction in patient symptomatology, conjunctival injection, and less than a two-step incrementation in patient satisfaction level. The treatment was considered “worsened” in case of two or more step increments in conjunctival injection and patient symptoms, and two or more step reductions in patient satisfaction level.

### Statistical analysis

Data was analyzed using SPSS for Windows (version 20.0). Qualitative data variables were expressed as frequency and percentage, while quantitative data variables were expressed as mean, standard deviation, and median. Wilcoxan-Mann-Whitney U and Chi-square tests were used to compare variables between the two groups. The confidence interval was 95%, and a p-value < 0.05 was considered statistically significant.

## Results

In this study involving 1328 patients diagnosed with acute conjunctivitis, we observed a gender distribution of 71.4% males and 28.6% females, with a mean patient age of 29.13 years (±7.15 years).

Conjunctivitis cases were categorized as pseudomembranous (42.9%) and non-membranous (57.1%). The most common symptoms at presentation included redness (99.47%), watering/discharge (75.30%), lid edema (54.82%), ocular discomfort/mild ocular pain (54.19%), foreign body sensation/grittiness (38.50%), and photophobia (30.72%).

**Graph 1:**
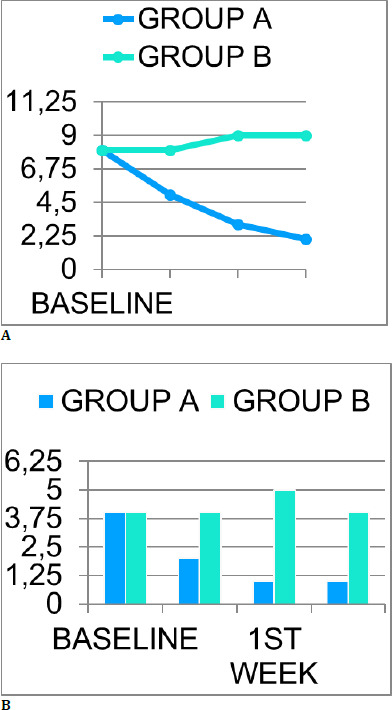
**A**. Comparison of reduction in ocular signs and symptoms between group A and group B **B**. Comparison of patient satisfaction level between group A and group B

The primary cause of conjunctivitis was adenoviral infection in conjunction with enteroviral infection with or without secondary staphylococcal conjunctivitis. Treatment outcomes indicated that Group A patients achieved successful treatment, with 76.05% (n=505) demonstrating improvement within an average of 2.6 days (±0.51 days). In contrast, 62.65% (n=416) of Group B patients exhibited recovery, with an average recovery time of 7.5 days (±1.1 days), which was found to be statistically significantly less than that of Group A (p >0.05). Notably, 13% of Group B patients developed recalcitrant conjunctivitis but responded well to treatment with a 1% betadine wash. **Table 2** summarizes all these findings. **Graphs 1A** and **B** illustrate the differences in ocular signs, symptoms, and patient satisfaction levels between Group A and Group B. Additionally, complications were observed in Group B patients, with 34.04% experiencing subconjunctival hemorrhage and 6.02% developing superficial punctate keratitis. No adverse reaction to LOS-pI was seen post-administration, and even on subsequent follow-ups.

**Table 2:** Conjunctivitis classification, presenting symptoms, and treatment outcomes

PARAMETER	MEAN±SD*/PERCENTAGE
**CONJUNCTIVITISTYPE**	
**-Pseudomembranous**	42.9%
**-Nonmembranous**	57.1%
**COMMONSYMPTOMSATPRESENTATION**	
**-Redness**	99.47%
**-Watering/Discharge**	75.30%
**-LidEdema**	54.82%
**-Oculardiscomfort/Mildocularpain**	54.19%
**-Foreignbodysensation/Grittiness**	38.50%
**-Photophobia**	30.72%
**TREATMENTOUTCOMES**	
**GroupA(Completelyresolved)**	76.05%
**GroupB(Completelyresolved)**	62.65%
**AVERAGETIMEFORCOMPLETERESOLUTION**	
**GroupA**	2.6±0.51days
**GroupB**	7.5±1.1days
**COMPLICATIONSINGROUPBPATIENTS**	
**-Sub-conjunctivalhemorrhage**	34.04%
**-Superficialpunctatekeratitis**	6.02%

* SD=Standard deviation

## Discussion

A widespread ocular ailment, conjunctivitis presents a multifaceted challenge for ophthalmologists and patients. This common condition management can be inconvenient, resulting in patient discomfort and frustration for healthcare providers. It is further exacerbated by the financial burden incurred through repeated clinic visits, the cost of topical medications, lost workdays, and diminished productivity. In this “instant” lifestyle era, the urgent need for rapid symptom relief cannot be overstated. Our study strived to address this issue by investigating the efficacy of a one-time, outpatient betadine eye wash as an adjunctive therapy for acute epidemic conjunctivitis, spanning both membranous and non-membranous cases. While adenovirus is traditionally known as the primary culprit behind conjunctivitis epidemics, our investigation into the 2023 outbreak revealed a distinctive viral co-infection involving adenovirus and enterovirus [3]. Due to this co-infection, the disease course was observed to be more severe and protracted, accompanied by complications, notably subconjunctival hemorrhages. Our approach encompassed conventional treatment alongside LOS-pI wash for patients in group A, while group B patients received traditional therapy alone.

Earlier research has demonstrated the effectiveness of 5% povidone-iodine for adenovirus conjunctivitis [8]. However, our study’s inclusion of bacterial conjunctivitis cases and a more tolerable 1% betadine solution use, sets it apart. Our study showed that even one-fifth concentration of betadine is effective for complete resolution of recalcitrant infectious conjunctivitis. Also, 5% betadine may cause conjunctival goblet cell damage, while 1% betadine is well-tolerated.

In a comprehensive pediatric study involving 459 children with conjunctivitis, 1.25% povidone-iodine eye drops proved as effective as traditional antibiotic drops for bacterial conjunctivitis and exhibited a slight superiority against chlamydia [9]. What is important to note is that they were equally effective against viral conjunctivitis [9]. In contrast, our study revealed that 1% povidone-iodine was efficacious against both viral and bacterial conjunctivitis.

An interesting finding in our study was that the complete resolution of the disease also occurred in most of the patients in group B, meaning 62.65%. However, it took nearly a week to reach that stage. Complete resolution of the disease within 2-3 days in group A patients showed significant effectiveness of LOS-pI wash in reducing the duration of the disease. Also, 34.04% of patients in group B progressed to sub-conjunctival hemorrhages and 6.02% of patients developed superficial punctate keratitis, however, none of the patients in group A experienced complications, in any form.

Since the 1950s, povidone-iodine has been a formidable compound boasting broad-spectrum antimicrobial properties [10]. Its mode of action involves transporting free iodine directly to the surface of target cells, oxidizing essential cellular constituents, and inactivating crucial viral enzymes [11,12]. This multifaceted mechanism underpins its effectiveness against many pathogens including bacteria and viruses [10].

Therefore, low concentrations of povidone-iodine use in the form of one-time eye wash, for all cases of viral, and bacterial conjunctivitis, can substantially reduce disease duration, and hence, the suffering of the patients.

No adverse reaction to betadine, post-administration, was noted in our study. It was well tolerated when administered under one drop of topical anesthesia. A significant reduction in complication rate was also observed in patients who were administered LOS-pI wash on presentation.

The importance of LOS-pI wash administering at the initial stage only lies in the fact that povidone-iodine can inactivate the virus cells in their extracellular stage itself, thereby, preventing them from infecting a larger number of cells [10-14]. The authors hypothesize that due to this reason, a single wash with betadine is sufficient to reduce disease duration and prevent secondary infections and complications. The concentration of free iodine varies between a 10% betadine solution, where it is 5 parts per million (ppm), and a 0.1% solution, where it is 24 ppm [13,14]. Consequently, the microbicidal effect sets in more rapidly with the 0.1%-1% povidone-iodine solution, taking just 15 seconds, compared to the 2.5%-10% povidone-iodine solution, which requires 30-120 seconds [13,14]. Therefore, a low concentration of povidone-iodine solution is preferable as it is well tolerated and more efficient.

The core strength of our study lies in its substantial sample size. To our knowledge, no similar previous research has been conducted on such a larger scale. Consequently, we advocate for further large-scale studies to delineate treatment protocols tailored for conjunctivitis epidemics. These endeavors will mitigate the economic burden associated with this condition and serve as a critical strategy in ensuring treatment accessibility, particularly in regions where medication shortages can cripple healthcare responses during outbreaks.

## Conclusion

Our research contributes valuable insights into epidemic conjunctivitis management, using one-time daycare low-concentration betadine eye wash, offering a promising approach for more efficient and cost-effective treatment in both epidemic and non-epidemic scenarios. Further studies in this direction could revolutionize the approach and treatment of this common ocular condition.
